# Age and Gender Perspectives on Social Media and Technology Practices during the COVID-19 Pandemic

**DOI:** 10.3390/ijerph192113969

**Published:** 2022-10-27

**Authors:** Mary Chidiac, Christopher Ross, Hannah R. Marston, Shannon Freeman

**Affiliations:** 1 Center for Technology Adoption for Aging in the North (CTAAN), University of Northern British Columbia, Prince George, BC V2N 4Z9, Canada; 2 Health and Wellbeing Strategic Research Area, School of Health, Wellbeing, Education and Language Studies Social Care, The Open University, Walton Hall, Milton Keynes MK7 6AA, UK; 3 School of Nursing, University of Northern British Columbia, Prince George, BC V2N 4Z9, Canada

**Keywords:** aging, interdisciplinary research, social connections, pandemic, health research, social technology, older people, social relationships, COVID-19, loneliness, isolation

## Abstract

Few studies have examined social media and technology use during the COVID-19 pandemic in Canada. Therefore, the main research question and objective of this study was to examine similarities and differences in the influences of mobile technology and social media use on Canadians among different age groups and across gender during the COVID-19 pandemic. From June through October 2021, 204 persons completed a 72-item online survey. Survey questions encompassed COVID-19 pandemic experiences and technology use. Standardized measures including the Psychological Wellbeing measure, eHeals, and the UCLA V3 Loneliness scale were collected to examine the psychological influences of the COVID-19 pandemic. Findings showed that males under 50 years were most likely to self-isolate compared to the other demographic results of the study. Males reported using technology less than females but were more likely to report using technology to share information regarding COVID-19. Respondents under 50 years were also more likely to use smartphones/mobile phones as their most used mobile technology device, whereas respondents over 50 were more split between smartphones/mobile phones and computers/tablets as their most used device. Males scored higher on the UCLA loneliness scale and lower on the Psychological Wellbeing sub-scores compared to females. Further research should explore additional demographics in relation to broader aspects of digital skills across different age groups.

## 1. Introduction

For many people, the use of technologies to access the Internet and social media platforms (i.e., computers, tablet, smartphones) have become integral to social connections in daily life [1]. Globally, the WHO estimates that approximately one billion individuals experience need for support from assistive technologies; however, access remains fragmented in part due to limited access and financial and policy barriers [2,3]. For those who are able to access technologies, they can play an important role in enhancing quality of life, and wellbeing, and can provide opportunity to facilitate shared learnings and positive interactions [4,5]. As such, it is worth exploring the influence technology may have on persons’ emotional and physical health and wellbeing during the COVID-19 pandemic.

During the COVID-19 pandemic lockdown, the feelings of social isolation and loneliness were common, and many persons substituted social media and ‘digital connections’ via technology to mitigate the feeling of being isolated [6,7]. This provided the opportunity to examine to what extent technology can reduce social isolation and loneliness for individuals during the COVID-19 pandemic. Beyond potentially reducing social isolation, there may be additional influences, behaviours, and usability experiences of engaging with technologies and social media platforms between community COVID-19 groups, individuals, and social networks.

Previous research examining technology and social media platforms has ranged from examining online forums among employees in organizations [8] to how technology and social media influences relationships between others [9], and even looking at the influence of social media on mental health and psychological wellbeing [10,11]. Kothari noted the positive influences of technology, pointing out its connective properties and the way it can be integrated into education [12]. Additional studies have ascertained the negative influences that technology and social media can have on people, especially on adolescent development [13], and how it can negatively influence health behaviors [14]. Abbas and Mahon explored the gendered difference in how males and females engage with social media; some studies found differences in how each gender uses and engages with social media, with many of such uses associated with concepts of body image and plastic surgery [15,16,17,18,19]. When examining problematic Internet use during the COVID-19 pandemic, Deutrom found that life satisfaction and age were positively associated with loneliness among adults working from home in the UK [20]. Furthermore, using technology as a means of accessing and using the Internet and social media platforms to facilitate information sharing is becoming an integral part of daily life [21,22].

Despite all this information on social media and its influences on various disciplines such as human computer interaction (HCI), communication, and media studies, there is comparatively very little information on the influence of the COVID-19 pandemic on technology and social media use in Canada. This is partially because the COVID-19 pandemic is not over at the time of writing, and much of the current research surrounding COVID-19 focuses on health and the public perceptions of government action and psychological/mental health implications of the pandemic [23,24,25,26,27].

For over a year, most people have had to adapt their regular social interactions to incorporate social distancing mandates, remote or working from home practices, and the reduction in ‘in-person’ leisure activities. Given previous studies identifying gendered differences in technology and social media use, additional examination of the presented data focuses on the age and gender differences pertaining to how COVID-19 has influenced general technology use [23,24]. With this in mind, our research objectives were to examine how technology and social media use influenced social connections and information sharing during the COVID-19 pandemic, and how this use has been interconnected with respective pandemic-related policies, including self-isolation and social distancing. Finally, utilizing the UCLA V3 loneliness scale, we examined how COVID-19 has affected the overall feelings of loneliness and how that may influence technology use across gender and age cohorts.

The work and findings presented here contribute to the fields of gerontechnology, gender studies, and gerontology as we specifically focus on technology and social media use by male and female respondents who are utilizing technology in later life. All of these are areas of continual growth pertinent to literature, sharing knowledge, and practices.

## 2. Materials and Methods

### 2.1. Survey

This study and its authors are active members of a larger international consortium of researchers surveying Technology, Social Connections, Loneliness and Leisure Activities during COVD-19 [28]. Methods of the global study have been previously reported [28].

In brief, the Canadian version of the survey, developed as part of wave-2 of the consortium data collection, was based on the global study methods, and included language adapted specific for North Americans. The Canadian version of the survey consisted of 72 questions organized into 8 sections, adapted from the global study method’s initial 65-question survey.

These sections consisted of demographics, social media use, and COVID-19 related questions, social networks/assistants/emergency systems, and older adult service usage assessment. In addition, there were three standardized measures integrated into the survey: the Psychological Wellbeing measure, the electronic health literacy measure (eHeals), and the UCLA loneliness scale (V3). The Psychological Wellbeing measure is a reliable questionnaire and was chosen as an effective measure of the six subcategories of wellbeing with a Likert scale: autonomy, environmental mastery, personal growth, positive relationships with others, purpose in life, and self-acceptance [29]. The eHeals measure is an eight-item questionnaire used to measure electronic health literacy, including the skills of finding health information online and its application to real life [30]. To measure the concept of loneliness during the COVID-19 pandemic, the UCLA loneliness scale was used as a valid and reliable measure of loneliness across populations during the pandemic using a Likert scale for its 20 items [31]. It is worth noting that the global methods talk of prospective participants, but this paper is based on a set of completed survey data.

### 2.2. Data Collection

Data collection occurred via the Survey Monkey online platform [32]. The survey link was shared on social media platforms (Facebook and Twitter), online, and through email mailing lists. Additionally, the link was accessible via a webpage on the Centre for Technology Adoption for Aging in the North (CTAAN) website (ctaan.ca). Data collection took place from June to October 2021. Study information was shared throughout the region of northern British Columbia with organizations that were likely to be supportive of sharing the survey information and link (e.g., older adult community organizations, public library). Completion of the survey took approximately 20 mins. Respondents confirmed they provided informed consent prior to starting the survey.

### 2.3. Data Analysis

Data analyses were conducted using SPSS version 27 [33]. Univariate and bivariate analyses examined survey response variables. Executing bivariate analyses aimed to identify associations between age, gender, and technology use during the COVID-19 pandemic. Descriptive (frequencies) analysis was conducted on key variables in the study. Inferential analysis included t-tests and chi-square tests depending on variable type.

## 3. Results

A total of 204 people completed the survey. Of the survey respondents, 77.8% were under the age of 50 years. Two-thirds of the survey respondents self-identified as female, and nearly half of the survey respondents reported having to self-isolate during the COVID-19 pandemic (Table 1).

**Figure 1:**
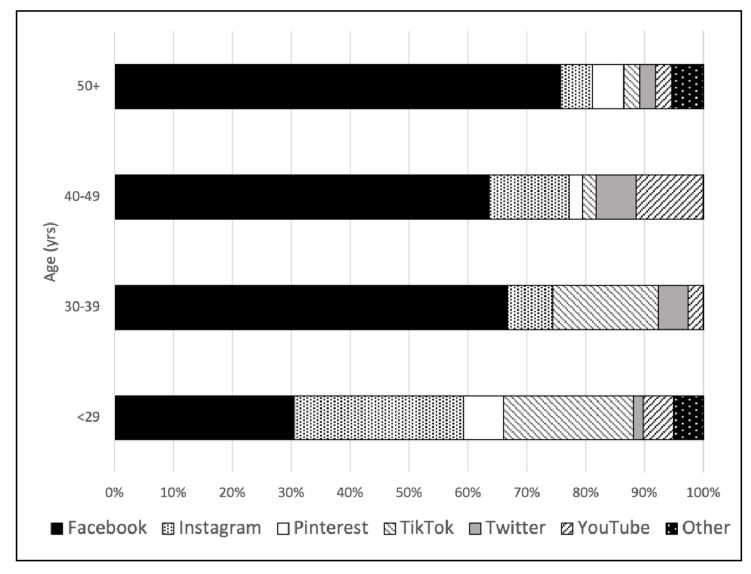
Social media platform use during the COVID pandemic by age among Canadian survey respondents; June 2021–October 2021 *(n* = 179).

Most survey respondents owned a computer (95.9%) and had home Internet access (97.9%), and most respondents primarily used a smartphone or mobile phone to access the Internet (81.1%). Facebook was the most dominant social media platform (55.2%) with all the other social media platforms being more evenly distributed across the sample (Figure 1). Moreover, 75% of respondents answered ‘yes’ to sharing information on COVID-19, and 43.5% of respondents were part of a COVID-19 support group.

Survey respondents under the age of 50 were more likely to be employed compared to those aged 50 years and over (89% vs. 52.3%; *p* < 0.001). Findings also ascertained the number of respondents who self-isolated during the COVID-19 pandemic was split: half of the respondents were aged under 50 years (51% No vs. 49% Yes), while the respondents aged 50 years and over showed a less even split, with one-third of the sample reporting having to self-isolate (65.9% No vs. 34.1% Yes) (Table 2). Most respondents under the age of 50 years reported more smartphone usage compared to laptop/tablet usage (89.1% vs. 10.9%; *p* < 0.001; Table 2), whereas the use of a smartphone or a computer or tablet was more evenly split in the 50+ years group (44.2% vs. 55.8%; *p* < 0.001; Table 2).

Regarding technology use, the sample who identified as being under the age of 50 showed they were more likely to engage in technology using a phone more so than respondents aged 50 years and over (89.1% vs. 55.8%; *p* <0.001). Respondents over the age of 50 years were more likely to report using Facebook compared to respondents under 50 years (OR 3.03CI 1.33–6.87; 75.7% vs. 50.7%; *p* = 0.006). Despite this difference, respondents under the age of 50 years were 2.48 times as likely to engage with online COVID-19 support groups compared to the respondents who were 50 years and over (48.9% vs. 27.9%; *p* = 0.02) (Table 2).

Data analysis also showed that male respondents were more likely to self-isolate than female respondents (64.1% vs. 36.6%; *p* < 0.001; Table 3), while female respondents were more likely to share information daily on social media than males (80.0% vs. 44.1%; *p* < 0.001). A significant relationship was noted between gender and both COVID-19 support groups and assistance during the pandemic. Male respondents reported a higher frequency of seeking out COVID-19 assistance (65.0% vs. 28.6%; *p* < 0.001; Table 3) and online support groups (59.3% vs. 36.3%; *p* = 0.003; Table 3), whereas respondents under the age of 50 years showed higher engagement in COVID-19 assistance (45.5% vs 25.0%; *p* = 0.01) and COVID-19 online support groups (48.9% vs 27.9%; *p* = 0.02; Table 2). There was no significant relationship observed between gender and employment status, gender and the most used digital device, or age and social media frequency (Table 2 and Table 3).

The UCLA loneliness scale showed a higher mean score for males compared to females (M = 47.79 vs. M = 43.59; t = −2.34, *p* < 0.05) (Table 4). The personal growth sub-score of the Psychological Wellbeing measure showed the biggest difference in mean score for males compared to females, with males having a lower mean compared to females (M= 16.84 vs. 14.2; t = 4.69 (*p* < 0.001). Furthermore, the other sub-scores showed males having overall lower mean scores compared to females, most notably with positive relationships with others (M = 12.37 vs. M = 14.55), purpose in life (M = 12.76 vs. 15.41), and self-acceptance (14.22 vs. 15.66). There was no significant difference in means for autonomy, environmental mastery, or the eHeals scores.

For age (Table 5), the UCLA loneliness scale showed a higher mean score for respondents under the age of 50 years in comparison to respondents over 50 years (M = 46.19 vs. M = 39.0, *p* < 0.001). The Psychological Wellbeing sub-score purpose in life showed the biggest difference in mean scores, with respondents under 50 years scoring lower than respondents over 50 years (M = 13.84 vs. M = 17.06, *p* <0.001). For the other sub-scores, respondents under 50 years tended to score lower than respondents over 50 years, particularly with self-acceptance (M = 14.67 vs. 17.16) and environmental mastery (M = 13.63 vs. M = 16.1). There was no significant difference in mean scores for the eHeals measure between age groups.

## 4. Discussion

The COVID-19 pandemic has forced the world to change the existing norms and routines associated with daily activities by adapting to, and continuing, social distancing through fewer in-person activities, such as working from home or virtual social practices. Although technology was a factor in a pre-pandemic society, the technology discourse has shifted from having more leisure connotations to being a necessity for everyday life. Through this shift in technology use, it is important to consider the changes in technology use that the COVID-19 pandemic has brought about.

This study examined technology engagement during the COVID-19 pandemic. We found differences between age and gender comparisons, as well as within the age and gender variables separately. Overall, the most notable result was that the highest levels of self-isolation during the COVID-19 pandemic were from male respondents aged 50 years and under. Consequently, this age group and gender category also reported the highest levels of loneliness according to the UCLA loneliness scale. Regarding age specifically, this finding supports previous data pertaining to the influence of the COVID-19 pandemic on loneliness in younger adults, as well as recent research showing greater levels of loneliness among younger adults during the COVID-19 pandemic in North America [27,34,35,36]. However, these findings also differ from other studies that ascertained higher levels of loneliness and depression among older adults [37,38].

Furthermore, this study found that females across all age groups reported the highest levels of social media usage. This finding adds to a mixed perspective on gender and social media, with some studies supporting our findings on gender and social media, whereas other studies found that males use social media more than females [39,40]. In this study, data shows females used social media the most, while males under 50 years reported using COVID-19 online support groups and assistance the most. This suggests a connection between self-isolation, loneliness, and seeking out supports. This finding and connection between self-isolation and higher levels of loneliness is underpinned by previous research [41], as supports have proven to be instrumental in helping people with their mental health; it would stand to reason that taking those supports away via self-isolation would cause higher levels of loneliness.

In examining the data between age and gender in relation to technology use, the lowest level of engagement came from the male sample aged 50 years and over (Table 6). This finding suggests males aged over 50 years were the least likely to use technology and social media. However, all the males aged over 50 years in our sample did report using social media daily. This discrepancy does not point to the frequency of technology use in male respondents over 50 years, but instead suggests that male respondents still use social media regularly, are less likely to engage with online surveys, and are instead more likely to engage with news and financial aspects via the Internet [42].

Female respondents in both age categories reported the highest levels of social media engagement, which is in line with previous studies [43]. Despite this, we found that male respondents under 50 years old reported the highest usage of online COVID-19 support groups and assistance. This difference is likely due to males under 50 years old also having the highest levels of self-isolation and the highest levels of loneliness in the sample. This contrasts with recent research pertaining to the COVID-19 pandemic in Canada, which ascertained that young adult females had the greatest levels of psychological distress during the pandemic [27]. Furthermore, it is worth noting that this previous research did not take self-isolation into account and had a greater focus on economic disparity, which we did not examine in this study [27]. Moreover, these points could suggest that self-isolating during the COVID-19 pandemic could lead to higher levels of loneliness, which in turn could lead to higher levels of support engagement. This difference is supported by female respondents over 50 years scoring significantly higher on the psychological wellbeing measures, which could also present a reason why they did not seek out supports as often as the male respondents under 50 years old. Despite these differences, both female and male respondents across the age groups all reported a high frequency of sharing COVID-19 information on social media during the pandemic. This points to COVID-19 information sharing being independent of self-isolation or loneliness/psychological wellbeing, and further supports our finding that self-isolation leads to a higher instance of seeking out supports.

Among the differences in age, one notable finding was that there was no significant difference identified from the eHeals scores by age group [30]. This finding supports previous research challenging the notion that older adults are less likely to use technology compared to younger generations and supports the positive experiences that older adults have with online health education [44,45].

In terms of how the different age groups engage with technology, Facebook proved to be the most popular social media platform among the respondents over 50 years, in comparison to the respondents under 50 years old. This also supports previous research pertaining to Facebook being the most popular social media platform [46]. This notion could be due to Facebook being one of the first social media platforms developed, and thus it may be a more familiar platform for people since it has been around the longest.

Furthermore, the results suggest that older adults engaged with social media using either a smartphone or a computer, whereas the younger age group was more likely to use a smartphone. Like social media, this difference may relate to familiarity with devices that people have used the most throughout their life. Computers have been used far more than smartphones and tablets because of how long computers have been accessible to users. With this in mind, it is probable such a device is preferred by older adults to engage with instead of a smartphone or a tablet. Although the split between smartphones and computers in the over-50 category suggests that many older adults are highly capable of using smartphones to engage with social media, previous research on the digital divide suggests this may decrease with older adults [46].

During the COVID-19 pandemic, the age difference in social media use observed suggests that persons aged less than 50 years were the most likely to seek out online supports. This could be due to the previously mentioned variables such as self-isolating and loneliness, as well as older adults scoring significantly higher on the psychological wellbeing sub-scores, and therefore having less of a theoretical need for COVID-19 supports. This is in line with previous research conducted in Canada, which found that young adults in Canada had great concern and psychological distress over the COVID-19 pandemic [19,30]. These mental health impacts could further explain why younger adults in our study had a higher instance of engaging with online supports. However, given that both age groups reported a daily use of social media and similar eHeals scores, it could be that COVID-19 supports were not as widely available, or perhaps as widely communicated, to older adults compared to persons under the age of 50 [47].

Comparing across gender, the data are consistent with those reported in the larger global survey dataset [28] suggesting that during the COVID-19 pandemic, male respondents experienced more loneliness while female respondents were more likely to be members of an online community support group(s) and to seek out assistance. This could be due to previous findings suggesting that males view technology as less of a leisure and more related to work and building connections [43]. Despite the male sample being smaller than the female sample, these trends were consistent. The significant association between self-isolation and gender continues through the digital practices of respondents pertaining to the social media and technology, and suggests gender differences in how respondents experienced COVID-19 and in how the COVID-19 pandemic influenced their use of technology.

### Strengths and Limitations

Considering limitations, the most notable is the small sample size, with 204 respondents to our survey. Therefore, we were limited in our ability to expand beyond descriptive bivariate analyses. These findings are suggestive of important patterns, which may be further investigated using the larger datasets in the future. Furthermore, we were limited in our ability to stratify by community size as most study respondents reported to live in suburban communities, with only 18.6% of respondents from metropolitan areas. Even though most respondents had access to the Internet and a computer, this demographic could have had an influence on the kinds of support groups or assistance programs the respondents were aware of, and it is a factor that should be considered when interpreting the findings. Moreover, it is important to consider limitations that stem from measuring human behaviors, such as biases (potential social desirability), and that scaled measures and surveys can only be effective in measuring human behavior. A final limitation readers should consider is within the data collection. Our survey was only made available in an online format. Thus, only participants who had Internet access were able to participate. This could have influenced the results in that only technology users were considered in the data by nature of the administration of the survey, which could present a reason for the lower frequency of participants over the age of 50.

The findings from this work have future implications for policy in Canada and the need for including greater support and assistance programs in different environments, as well as specific targeting of audiences [48]. Future considerations, policies, and programs should consider appropriate support and tailored information targeting males under the age of 50 years. The work presented here highlights that male respondents are the group that both engaged in COVID-19 supports the most and appeared to be the most in need of such resources. Therefore, the implication is that future programs and support should ensure COVID-19 supports and assistance are more accessible and available across ages and genders, especially as we transition into a post-pandemic society. Targeted support and programs specifically for men under the age of 50 years may require alternative approaches outside the regular routes and pathways, focusing on male-only groups (both online and offline). In addition, for appropriate information to be retrieved, future work is needed to fully understand what they would need and how to reach these younger and middle-aged men. For example, advertisements of appropriate programs and support may be distributed on social media groups, gyms and fitness environments, sports bars, and arenas. However, conducting co-production workshops with this specific target audience would identify clearer access routes in the long term.

## 5. Conclusions

This paper presents a portion of data collected from a larger, international, multi-sited study focusing on social isolation and technology use during the pandemic from the perspective of gender and age. The findings presented contribute to several disciplines including gender studies, media communications, and gerontechnology. Furthermore, these findings should inform health and wellbeing policies and programs in Canada because, as our paper shows, a high proportion of men under the age of 50 years sought out COVID-19 support and resources. Although we ascertained men in this data sample used technology less frequently than females, their use of technology was for a specific purpose.

## Figures and Tables

**Table 1:** Demographics.

Demographics		All Respondents 100% *(N =* 204)	Missing, % (*n*)
Age (years)			1.0 (2)
	Under 50	77.7 (157)	
	50 and over	22.3 (45)	
Gender			1.5 (3)
	Male	32.3 (65)	
	Female	67.7 (136)	
Education			0.5 (1)
	High School or Less	15.8 (32)	
	Some College/University	26.6 (54)	
	College Diploma	16.7 (34)	
	Bachelor’s Degree	24.1 (49)	
	Master’s Degree or Higher	16.7 (34)	
Self-isolation due to COVID-19			1.5 (3)
	No	54.7 (110)	
	Yes	45.3 (91)	
Marital status			1.0 (2)
	Not married	38.1 (77)	
	Married/Common-law	61.9 (125)	
Employment status			1.5 (3)
	Not Working	18.9 (38)	
	Working	81.1 (163)	
Community size			
	<1000 (Rural)	6.9 (14)	
	1001–29,999 (Small Town)	23.5 (48)	
	30,000–99,999 (Suburban)	51.0 (104)	
	>100,000 (Metropolitan)	18.6 (38)	
COVID-19 support group member			8.8 (18)
	No	56.5 (105)	
	Yes	43.5 (81)	
Own a computer			4.4 (9)
	No	4.1 (8)	
	Yes	95.9 (187)	
Frequency of computer use			6.9 (14)
	1–3 times/week	25.8 (49)	
	Daily	74.2 (141)	
Home Internet access			4.9 (10)
	No	2.1 (2.0)	
	Yes	97.9 (190)	
Digital devices owned			1.5 (3)
	Smartphone	30.9 (159)	
	Desktop PC/Laptop	28.6 (147)	
	Tablet	22.6 (116)	
	Mobile phone	17.9 (92)	
Social media platforms used			5.9 (12)
	Facebook	26.0 (159)	
	Instagram	16.2 (99)	
	YouTube	15.7 (96)	
	Pinterest	11.3 (69)	
	TikTok	10.6 (65)	
	Twitter	10.5 (64)	
	LinkedIn	7.8 (48)	
	Other	1.0 (6)	
	None	1.0 (6)	
Most used social media platform			11.3 (23)
	Facebook	55.2 (100)	
	Instagram	16.0 (29)	
	TikTok	12.7 (23)	
	YouTube	5.5 (10)	
	Twitter	3.9 (7)	
	Pinterest	3.9 (7)	
	Other	2.8 (5)	
COVID-19 information sharing on device			13.7 (28)
	No	25.0 (44)	
	Yes	75.0(132)	
COVID-19 information sharing frequency			36.8 (75)
	Daily	7.8 (10)	
	Weekly	92.2 (119)	
COVID-19 assistance *			
	No	59.8 (113)	7.4 (15)
	Yes	40.2 (76)	

* *p* < 0.05.

**Table 2:** Key variables stratified by age group (*n* = 199).

	Age Group %, (*n* = 199)		Odds Ratio (95% CI) ^1^	Missing%, (*n*)
	<50 % *(n)*	≥50 % (*n*)		
Gender ****				2.5 (5)
Female	61.3 (95)	88.6 (39)	4.93 (1.84–13.2)	
Male	38.7 (60)	11.4 (5)	1.00	
Employment status ***				2.5 (5)
Not Working	11.0 (17)	47.7 (21)	1.00	
Working	89.0 (138)	52.3 (23)	7.41 (3.41–16.12)	
Marital status				1.5 (3)
Married/commonlaw	59.0 (92)	73.3 (33)	1.00	
Not married	41.0 (64)	26.7 (12)	1.91 (0.92–3.98)	
Self-isolation *				2.5 (5)
No	51.0 (79)	65.9 (29)	1.00	
Yes	49.0 (76)	34.1 (15)	1.86 (0.93–3.74)	
Most used device ***				6.9 (14)
PC/Tablet	10.9 (16)	44.2 (19)	1.00	
Phone	89.1 (131)	55.8 (24)	6.48 (2.93–14.35)	
Social media frequency *				11.8 (24)
Daily	64.3 (92)	81.1 (30)	1.00	
Weekly	35.7 (51)	18.9 (7)	2.38 (0.98–5.79)	
Most used social media platform **				12.3 (25)
Facebook	50.7 (72)	75.7 (28)	1.00	
Other	49.3 (70)	24.3 (9)	3.03 (1.33–6.87)	
COVID-19 information sharing on device				14.7 (30)
No	27.2 (37)	15.8 (6)	1.00	
Yes	72.8 (99)	84.2 (32)	1.99 (0.77–5.16)	
COVID-19 information sharing frequency				37.3 (76)
Daily	5.9 (6)	14.8 (4)	1.00	
Weekly	94.1 (95)	85.2 (23)	2.75 (0.72–10.57)	
COVID-19 support group member *				9.8 (20)
No	51.1 (72)	72.1 (31)	1.00	
Yes	48.9 (69)	27.9 (12)	2.48 (1.18–5.21)	
COVID-19 assistance *				8.3 (17)
No	54.5 (78)	75.0 (33)	1.00	
Yes	45.5 (65)	25.0 (11)	2.05 (1.11–3.81)	

^1^ (CI) Confidence interval. Significant differences by age group are denoted as follows: * *p* < 0.05, ** *p* < 0.01, *** *p* < 0.001, **** *p* < 0.0001.

**Table 3:** Key variables stratified by gender (*n* = 201).

		Gender %, (*n* = 201)		Odds Ratio (95% CI) ^1^	Missing %, (*n*)
		Female, % (n)	Male, % (n)		
Age ***					2.5 (5)
	Under 50	70.9 (95)	92.3 (60)	1.00	
	50 and Over	29.1 (39)	7.7 (5)	4.93 (1.84–13.2)	
Employment status					2.9 (6)
	Not working	21.8 (29)	13.8 (9)	1.00	
	Working	78.2 (104)	86.2 (56)	1.74 (0.77–3.92)	
Marital status *					2.0 (4)
Married/commonlaw		56.3 (76)	72.3 (47)	1.00	
	Not married	43.7 (59)	27.7 (18)	2.03 (1.07–3.85)	
Self-isolation **					2.9 (6)
	No	63.4 (85)	35.9 (23)	1.00	
	Yes	36.6 (49)	64.1 (41)	3.09 (1.66–5.75)	
Most used device *					7.4 (15)
	PC/Tablet	21.3 (27)	11.3 (7)	1.00	
	Phone	78.7 (100)	88.7 (55)	2.12 (0.87–5.19)	
Most used social media platform					12.7 (26)
	Facebook	57.1 (68)	52.5 (31)	1.00	
	Other	42.9 (51)	47.5(28)	1.20 (0.64–2.25)	
Social media frequency **					12.3 (25)
	Daily	80.0 (96)	44.1 (26)	1.00	
	Weekly	20.0 (24)	55.9 (33)	5.08 (2.57–10.04)	
COVID-19 information sharing					15.2 (31)
	No	27.0 (31)	19.0 (11)	1.00	
	Yes	73.0 (84)	81.0 (47)	1.58 (0.73–3.42)	
COVID-19 information sharing frequency					37.3 (76)
	Daily	6.1 (5)	10.9 (5)	1.00	
	Weekly	93.9 (77)	89.1 (41)	1.88 (0.51–6.87)	
COVID-19 support group member ***					10.3 (21)
	No	63.7 (79)	40.7 (24)	1.00	
	Yes	36.3 (45)	59.3 (35)	2.56 (1.36–4.83)	
COVID-19 assistance ****					8.8 (18)
	No	71.4 (90)	35.0 (21)	1.00	
	Yes	28.6 (36)	65.0 (39)	4.64 (2.41–8.95)	

^1^ (CI) Confidence interval. Significant differences by gender are denoted as follows: * *p* < 0.05, ** *p* < 0.01, *** *p* < 0.001, **** *p* < 0.0001.

**Table 4:** Results of *t*-tests comparing males and females on standardized measures of loneliness (UCLA) eHealth literacy (eHeals) and wellbeing (Psychological Wellbeing).

Standardized Measures	Female		Male		t	Cohen’s d
	M	SD	M	SD		
UCLA loneliness sums *	43.59	10.69	47.79	8.76	−2.34	10.1
eHeals sums	32.79	6.5	30.94	5.99	1.72	6.33
Autonomy *	15.31	3.33	14.11	2.74	2.29	3.14
Environmental mastery	14.14	3.84	13.96	2.76	0.31	3.51
Personal growth ***	16.84	3.36	14.2	3.44	4.69	3.39
Positive relationships with others ****	14.55	3.46	12.37	3.27	3.83	3.4
Purpose in life ****	15.41	3.52	12.76	3.56	4.51	3.53
Self-acceptance *	15.66	3.52	14.22	3.25	2.5	3.43

Note: Significant differences by gender are denoted as follows: * *p* < 0.05, ** *p* < 0.01, *** *p* < 0.001, **** *p* < 0.0001.

**Table 5:** Results of *t*-tests comparing survey respondents aged under 50 and aged 50 and over on standardized measures of loneliness (UCLA) eHealth literacy (eHeals) and wellbeing (Psychological Wellbeing).

Standardized Measures	Aged < 50		Aged ≥ 50		t	Cohen’s d
	M	SD	M	SD		
UCLA loneliness sums ****	46.19	9.74	39.0	10.6	3.49	9.92
eHeals sums	31.81	6.33	33.41	6.8	−1.25	6.43
Autonomy *	14.73	3.02	16.26	3.25	−2.49	3.06
Environmental mastery ****	13.63	3.2	16.1	3.88	−3.65	3.34
Personal growth ***	15.43	3.65	17.81	2.72	−3.46	3.48
Positive relationships with others ***	13.3	3.52	15.72	3.13	−3.54	3.45
Purpose in life ****	13.84	3.63	17.06	3.02	−4.58	3.52
Self-acceptance ****	14.67	3.31	17.16	3.49	−3.76	3.34

Note: Significant differences by age group are denoted as follows: * *p* < 0.05, ** *p* < 0.01, *** *p* < 0.001, **** *p* < 0.0001.

**Table 6:** Key variables stratified by age and gender (*n* = 199).

	Age Group < 50% (*n* = 155)		Aged Group ≥ 50% (*n* = 44)		Missing %, (*n*)
	Female	Male	Female	Male	
Employment status					3.9 (8)
Working	90.3 (84)	86.7 (52)	47.4 (18)	80.0 (4)	
Not Working	9.7 (9)	13.3 (8)	52.6 (20)	20.0 (1)	
Marital status					2.5 (5)
Married/common-law	49.5 (47)	73.3 (44)	74.4 (29)	60.0 (3)	
Not married	50.5 (48)	26.7 (16)	25.6 (10)	40.0 (2)	
Self-isolation					3.9 (8)
No	63.4 (59)	31.7 (19)	61.5 (24)	100.0 (4)	
Yes	36.6 (34)	68.3 (41)	38.5 (15)	0.0 (0)	
Most used device					8.3 (17)
PC/Tablet	13.6 (12)	7.0 (4)	40.5 (15)	60.0 (3)	
Phone	86.4 (76)	93.0 (53)	59.5 (22)	40.0 (2)	
Social media frequency					13.2 (27)
Daily	80.2 (69)	40.0 (22)	78.1 (25)	100.0 (4)	
Weekly	19.8 (17)	60.0 (33)	21.9 (7)	0.0 (0)	
Most used social media platform					13.7 (28)
Facebook	49.4 (42)	52.7 (29)	81.3 (26)	50.0 (2)	
Other	50.6 (43)	47.3 (26)	18.8 (6)	50.0 (2)	
COVID-19 information sharing on device					16.2 (33)
No	33.8 (27)	16.7 (9)	9.1 (3)	50.0 (2)	
Yes	66.3 (53)	83.3 (45)	90.9 (30)	50.0 (2)	
COVID-19 information sharing frequency					37.7 (77)
Daily	3.6 (2)	8.9 (4)	11.5 (3)	100.0 (1)	
Weekly	96.4 (53)	91.1 (41)	88.5 (23)	0.0 (0)	
COVID-19 support group member					11.3 (23)
No	61.2 (52)	35.2 (19)	67.6 (25)	100.0 (5)	
Yes	28.8 (33)	64.8 (35)	32.4 (12)	0.0 (0)	
COVID-19 assistance					9.8 (20)
No	69.8 (60)	30.9 (17)	73.7 (28)	80.0 (4)	
Yes	30.2 (26)	69.1 (38)	26.3 (10)	20.0 (1)	

## Data Availability

We are still conducting analysis from this dataset as part of a large international study and we cannot provide the full dataset at present. Persons interested in accessing the data may contact the corresponding author.
